# Optimization of Phenolics Extracted from *Idesia polycarpa* Defatted Fruit Residue and Its Antioxidant and Depigmenting Activity *In Vitro* and *In Vivo*

**DOI:** 10.1155/2014/931269

**Published:** 2014-06-17

**Authors:** Yang Ye, Xiao-Shan Tang, Fang Chen, Lin Tang

**Affiliations:** National and Local Joint Engineering Laboratory for Energy Plant Bio-Oil Production and Application, Sichuan Key Laboratory of Resource Biology and Biopharmaceutical Engineering, Key Laboratory of Bio-Resources and Eco-Environment, College of Life Sciences, Sichuan University, Ministry of Education, Chengdu 610064, China

## Abstract

Extraction of phenolics from *Idesia polycarpa* defatted fruit residue was optimized by the maximization of the yield in total phenolics, using the response surface methodology. The optimized conditions were 50% ethanol, 5 h extraction time, 1 : 40 liquid to solid ratio, and 80°C extraction temperature. The experimental average total phenolics yield was 54.49 ± 4.26 mg/g. These antioxidant properties of phenolics were comprehensively analyzed for the first time. All the extracts not only demonstrated the significant free radical scavenging activities and metal chelating activity but also inhibited lipid, lipoprotein peroxidation and revealed reducing power activity. Ethyl acetate extraction (EAE) also inhibited mushroom tyrosinase activity and significantly increased the average skin-whitening index (L value) of the skin of C57BL/6 mice, indicating its potential use for skin hyperpigmentation in humans. The results of cell experiments showed EAE could strongly inhibit cellular tyrosinase activity, which had led to the decrease of melanogenesis in B16 mouse melanoma cells. Overall, EAE is an excellent natural antioxidant and depigmenting agent, which can be developed as a new food additive, medicine, and cosmetic.

## 1. Introduction

*Idesia polycarpa* is a deciduous tree of the Flacourtiaceae family and widly distributed. Oil content of* Idesia polycarpa* fruit is more than 40%. The oil is rich in various unsaturated fatty acids, such as linoleic acid and linolenic acid, and has been confirmed that is nontoxic and edible [1]. Hence, the oil has a good healthcare effect and can play a therapeutic role, which has been produced cooking oil in Asia for many years. Furthermore, there are lots of phenolics in fruit, such as idescarpin, which has the skin-whiting effect [2]. Idesin and salirepin extracted from defatted fruit residue have anti-inflammatory effect [3]. However, the fruit residue does not get reasonable utilization after oil extraction.

Reactive oxygen species (ROS) are critical to biological processes in most living organisms, which are continuously produced as a consequence of mitochondrial electron transfer processes or by-products of reactions catalyzed by various enzymes [4]. Excess amount of ROS, especially free radicals, can accelerate cells and tissue decomposition, affect the metabolic function, and cause different health problems [5, 6]. When excess free radicals are antagonized by antioxidants, numerous illnesses caused by free radicals and aging-related diseases could be prevented, such as cancer, arteriosclerosis, diabetes, and cardiovascular disease.

Compared to synthetic antioxidants such as BHA and BHT, there are no noticeable side effects of natural antioxidants. Therefore, natural antioxidants attract extensive attention worldwide [7, 8]. Many natural antioxidants, such as phenolics, have a strong antioxidant capacity and have been widely used in food, medicine [9], cosmetic [10], and so forth. For example, mulberry phenolics have outstanding skin-whitening effect [11]. For these reasons, phenolics have received considerable attention.

The skin color of humans and animals is primarily determined by the content of melanin; melanin cannot be synthesized without tyrosinase [12, 13]. Tyrosinase is a key enzyme of melanin synthesis* in vivo* with unique dual-catalytic function, which is bound up with the anile process of the human body. Overexpression of the tyrosinase gene severely caused hyperpigmentation disease, such as freckle, chloasma, and senile plaque [12]. Tyrosinase inhibitor could treat many current common hyperpigmentation diseases to whiten the skin by inhibiting activity of tyrosinase [14].

RSM is a commonly used data analysis, statistical approach for analyzing interactions among factors, exploring the relationships between the dependent variable and the independent variables and building an empirical model [15, 16]. It is usually employed to the optimization.

To the best of our knowledge, there are no reports on the extraction conditions and antioxidant activities and depigmenting activity of total phenolics extracted from* Idesia polycarpa* defatted fruit residue. Therefore, the extraction conditions were confirmed by using RSM. The extracts were analyzed by eight various antioxidant activity tests. Moreover, depigmenting activities of EAE* in vitro* and* in vivo* were analyzed in this study.

## 2. Materials and Methods

### 2.1. Fruit Samples

Fruits of* Idesia polycarpa* were collected from Guangyuan (Sichuan, China). After being placed in the shade to dry up to constant weight at room temperature, fruits were ground and sieved with a 40-mesh sieve. Before analysis, the oil of samples were removed by Soxhlet extraction for 5 h (petroleum ether as solvent). The defatted powder was stockpiled at −20°C.

### 2.2. Reagents

FC-reagent, catechin, sodium nitrite, TBA, Gallic acid, ABTS, DPPH, linoleic acid, *β*-carotene, BHT, phenanthroline, pyrogallol, VE, VC, MTT, arbutin, Triton X-100, tyrosine, L-Dopa, trypsin/EDTA, and HQ were purchased from Sigma Chemistry Co. (Beijing Agency, China). All other reagents were of analytical grade and purchased from Changzheng, Ltd. (Chengdu, China).

### 2.3. Preliminary Extraction Experiments

Before experimental design of the RSM procedure, we confirmed the most appropriate extraction solvent among ethanol, methanol, acetone, and water by using a liquid to solid ratio 1 : 20 (v : m); 1 g defatted powder was extracted at 50°C for 3 hours. Then, we chose the appropriate conditions (ethanol concentration, liquid to solid ratio, temperature, and time) to carry out a set of single-factor experiments. The first step of the experiment was to study the effect of a liquid to solid ratio on the extraction, by comparing five ratios (1 : 10 to 1 : 50): 1 g defatted powder was extracted with 80% ethanol at 50°C for 3 hours. The second step was to study the effect of extraction time in the range from 2 h to 6 h; 1 g sample was extracted with 80% ethanol at 50°C using a liquid to solid ratio of 1 : 20. The next step was to study the effect of ethanol concentration in the range from 40% to 80% (EtOH : H_2_O, v : v) and effect of extraction temperature (50°C to 90°C); the other conditions were consistent with the previous experiments.

### 2.4. Experiment Designs

The optimization of extraction parameters of phenolics from* Idesia polycarpa* defatted fruit residue was carried out by RSM. A four-factor, there-level Box-Behnken design (BBD) was inspired by the results of preliminary experiments. The design factors were the ethanol concentration (50%, 60%, and 70%; *X*_1_), the extraction time (4 h, 5 h, and 6 h; *X*_2_), the liquid to solid ratio (1 : 30, 1 : 40, and 1 : 50; *X*_3_), and the extraction temperature (70°C, 80°C, and 90°C; *X*_4_). The order of the experiments has been arrayed randomly. A second-order polynomial model was used to express the extraction as a function of the independent variables as follows:

(1) $$\begin{array}{c} Y=\alpha_{0}+\overset{4}{\underset{i=1}{\sum }}\alpha_{i}X_{i}+\overset{4}{\underset{i=1}{\sum }}\alpha_{ii}X_{i}^{2}+\overset{3}{\underset{i=1\;}{\sum }}\overset{4}{\underset{j=2}{\sum }}\alpha_{ij}X_{i}X_{j}, \end{array}$$

where *Y* represents the response variables; *α*_0_ is a constant; *α*_*i*_, *α*_*ii*_, and *α*_*ij*_ are the linear, quadratic, and interactive coefficients, respectively. *X*_*i*_ and *X*_*j*_ are the levels of the independent variables.

### 2.5. Fractional Extraction

Sample (1000 g) was extracted from the best conditions. Then, the crude extract was filtered and concentrated in a rotary evaporator. The yield of the product was 40.6% and the product was labeled CE. CE was dissolved in redistilled water in preference to fractional extraction using ethyl acetate, n-butyl alcohol in the order. After being removed solvents EAE, BAE, AF, and CE were full of nitrogen and stockpiled at −20°C until analyzed.

### 2.6. Total Phenolic Content

The method used to determine total phenolic content was on the basis of the FC method reported elsewhere [17] with proper modifications. Briefly, 1 mL extracts were mixed with 1.5 mL FC reagent and 10 mL 7% sodium carbonate solution was added 5 min later. The mixture was incubated at 30°C for 1 h with constant oscillation. The observance was gauged at a 765 nm wavelength. Gallic acid was used as a standard (0.002−0.025 mg/mL). The content of phenolics was expressed as mg Gallic acid equivalent/g of defatted fruit powder (mgGAE/g).

### 2.7. Total Flavonoid Content

Total flavonoid content was determined by aluminium chloride colorimetric assay [6] with some modifications. 1 mL extracts or standard (catechin) diluted with 11.5 mL 60% ethanol was added 0.7 mL 5% sodium nitrite. Next, the mixture was incubated for 5 min at room temperature, followed by addition of 0.7 mL 10% aluminium chloride, and 5 mL 1 mol/L sodium hydroxide was added 6 min later. The final volume was completed to 25 mL with 60% ethanol and the absorbance was measured at 510 nm. Total flavonoid content was expressed as mg catechins equivalent/g of defatted fruit powder (mgCaE/g).

### 2.8. Free Radicals Scavenging Activities

Currently accepted radicals scavenging experiments contain DPPH, ABTS, hydroxyl, and superoxide free radical scavenging experiments [18]. DPPH [19], ABTS [19], and hydroxyl free radicals [20] scavenging activities were measured strictly with the existing methods.

The superoxide radical scavenging activity (SOD-like activity) was measured by means of pyrogallol oxidation. Briefly, 3 mL 0.1 mol/L Tris-HCl buffer (pH 8.2), 0.2 mL 0.1 and 0.2 mg/mL of sample/standard (BHT and VC), and 3 mL 3 mmol/L pyrogallol solution were mixed, followed by twice dilution with water in order to complete a final volume of 10 mL. The absorbance was measured at 325 nm and defined as *A*_1_. In the first four minutes of absorbance measuring, each 30 s of the absorbance shown by the ultraviolet spectrophotometer was recorded. The gradient *k*_1_ was calculated according to the standard curve drawn by using these data, while the absorbance of 3 mL 0.1 mol/L Tris-HCl buffer (pH 8.2) and 3 mL 3 mmol/L pyrogallol solution without sample or standard was labeled as *A*_0_ under the same conditions, and those data of *A*_0_ were used to attain the gradient *k*_0_. SOD-like activity (%) = [(*k*_0_ − *k*_1_)/*k*_0_] × 100.

### 2.9. Reducing Power

Reducing power was determined strictly as reported [19]. The reducing power of each extract was determined as BHT equivalent at the same concentration.

### 2.10. Metal Chelating Activity

Metal chelating activity was assayed as reported [21]. Briefly, 1 mL samples or standard (EDTA) at five different concentrations (2, 4, 6, 8, and 10 mg/mL) was mixed with 2 mL 0.2% ferrous sulfate. After being incubated in a hot bath at 37°C for 30 min, the mixture was added with 0.5 mL 0.1% phenanthroline and incubated at 37°C for another 10 min and then centrifuged at 4000 rpm for 5 min. The absorbance of the upper solution was measured at 510 nm. The ferrous ion-chelating effect of the extracts was determined as EDTA equivalent.

### 2.11. *β*-Carotene Bleaching Test

Depending on the method of Tepe et al. [22], *β*-carotene bleaching test was measured with some modifications. 3 mL 0.1 mg/mL *β*-carotene prepared in trichloromethane and 40 *μ*L of linoleic acid were mixed with 400 *μ*L of Tween-40, and the trichloromethane was removed from the mixture by using the rotary evaporator. Then, the dry mixture was added with 200 mL of redistilled water and ultrasonic dissolved into an emulsion. 5 mL of the emulsion was mixed with 100 *μ*L 2 mg/mL extracts; 5 mL of the emulsion was mixed with 100 *μ*L 60% ethanol. The absorbance was measured at 470 nm and recorded every 20 min until the absorbance of the negative control dropped to 0.03. The mixture was incubated in a bath at 50°C during the period of absorbance measuring.

### 2.12. Inhibition of Lipoprotein Oxidation

The inhibition of yolk lipoprotein peroxidation was determined as reported in China [23]: 0.2 mL 4% suspension prepared in PBS (0.01 mol/L, pH 7.4), 0.2 mL extracts/standard (VE), 0.2 mL 25 mmol/L ferrous sulfate, and 1.4 mL of PBS were added and the mixture was incubated at 37°C for 15 min with constant oscillation. Then, 1 mL 10% TCA was added to the mixture to stand for another 10 min. After the mixture was centrifuged at 3500 rpm for 10 min, 2 mL of the upper was mixed with 1 mL of 0.8% TBA and incubated in boiling water for 15 min. After the mixture was water-cooled to room temperature, the absorbance was measured at 532 nm.

### 2.13. Mushroom Tyrosinase Activity

The assay was measured as described by Mapunya et al. [24] with some modification. Briefly, 40 *μ*L of sample, 80 *μ*L of 0.2 mol/L PBS (pH 6.8), and 40 *μ*L of mushroom tyrosinase (150 unit/mL) were mixed in a 96-well microplate at 37°C for 15 min. Then, the mixture was added with 40 *μ*L of substrate (L-tyrosine/L-Dopa) for further 20 min incubation. The absorbance of the mixture was measured at 475 nm. Arbutin and HQ were used as positive control.

### 2.14. Cell Culture

B16 mouse melanoma cells were obtained from the Chinese Academy of Sciences (CAS, Shanghai). The cells were cultured in DMEM medium containing 10% fetal bovine serum, 1 mmol/L sodium pyruvate, 2 mmol/L glutamine, 100 Units/mL penicillin, and streptomycin in a humidified 5% CO_2_/95% air controlled incubator at 37°C.

### 2.15. Cell Viability

MTT assays were used to determine the cell viability. Briefly, after cells were incubated with samples (1–1000 *μ*g/mL) for 48 h, 10 *μ*L MTT (5 mg/mL) was added to each well for further 4–6 h incubation. 100 *μ*L SDS prepared in 0.01 mol/L HCl was added to terminate the reaction and the absorbance of the dissolved formazan crystals was measured at 570 nm.

### 2.16. Measurements of Cellular Tyrosinase Activity

After 48 h of cell cultivation, the medium was discarded and the cells were added in 100 *μ*L 1% Triton X-100 at −80°C for 60 min. Then, the mixture was added with 50 *μ*L 0.1% L-Dopa at 37°C for further 2 h, following which the dopachrome formation was measured at 492 nm. Relative tyrosinase activity was obtained by dividing the enzyme activity of the reaction mixture with a sample of that passive control.

### 2.17. Determinations of Melanin Content

At the end of cell culture, the cells were collected and washed twice with PBS. The cells were homogenized in 0.05% trypsase-0.53 mmol/L EDTA. After centrifugation at 3000 rpm for 5 min, the supernatant was ruled out while the residue was added with 1 mol/L NaOH containing 10% DMSO for 2 h at 80°C. The absorbance was measured at 492 nm and the quantity of the cells was counted. Therefore, the relative content of melanin was calculated by OD_492_/cell quantity.

### 2.18. Determinations of Depigmenting Activity in Mice

Depigmenting activity was measured as described by Ding et al. [25] with little modification. The Institution Animal Care and Use Committee of Sichuan University approved this study. Six-week-old mice (C57BL/6), weighing around 30 ± 2 g, were obtained from Slac Laboratory Animal, Shanghai. Throughout the experiment, animals were housed in stainless steel cages in an air-conditioned room, and temperature was maintained at 25–27°C under the daylight cycle of 12 h. The animals were acclimatized for 7 d prior to the experiment. After shaving their hair, the animals were given 1 d rest. EAE or HQ was prepared by dispersing 1%, 2%, and 4% EAE or 4% HQ in water/propylene glycol/ethanol (6 : 3 : 1 v/v/v) with 1% menthol. A total of 40 mice were divided into five groups, and each group was smeared twice daily with 0.5 mL water/propylene glycol/ethanol with 1% menthol (control), 1% EAE, 2% EAE, 4% EAE, or 4% HQ. The applications have continued for 3 weeks, and the skin-whitening index (*L* value) was measured on the same skin area every day with Chromameter CR-10 (Konica Minolta, Osaka, Japan).

### 2.19. Statistical Analysis

Each test was performed in triplicate (the animal experiment was analyzed from 8 mice), and all results were expressed as the means plus-minus their corresponding standard deviation. Statistical analysis was measured by using the SPSS software (SPSS, IBM, version 19). The adequacy of the model was determined by evaluating the lack of fit, the coefficient of determination (*R* ^2^), and the *F*-test value obtained from the analysis of variance (ANOVA) that was generated. The significance of difference among means was determined by using one-way ANOVA and compared by Dunnett's multiple range test. The significance was determined by using *t*-test at 0.05 levels.

## 3. Results and Discussion

### 3.1. Analysis of Response Surface

**Figure 1:**
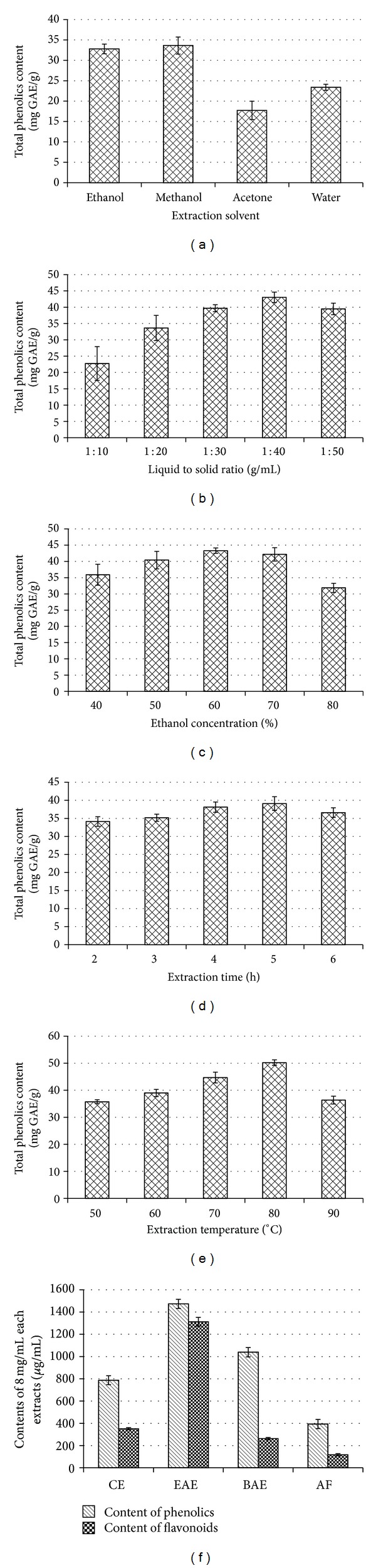
Effect of different extraction factors and total phenolics and flavonoids contents. ((a)–(e)) Effect of different extraction factors performed by single-factor test; (f) the total phenolics and flavonoids contents of all extracts at the same 8 mg/mL concentration. Each value is expressed as mean ± standard deviation (*n* = 3).

Although the difference between the extraction yields of ethanol and methanol was insignificant (*P* > 0.05, Figure 1(a)), methanol was more poisonous than ethanol; it was the reason why we had chosen ethanol as the extraction solvent.

The effect of ethanol concentration was determined by phenolic content shown in Figure 1(c). The yield of 60% of ethanol concentration was the highest, so the concentration of 60% was selected as the central value for the RSM. Similarly, the liquid to solid ratio 1 : 40, the extraction time 5 h, and the extraction temperature 80°C were chosen as the central value for the RSM.

29 of the designed experiments were conducted for optimizing the four individual parameters in the current Box-Behnken design.  Table 1 showed the experimental conditions and extraction yields, according to the factorial design. By using multiple regression analysis on the experimental data, the response variable and the test variables were related by the following second-order polynomial equation:

(2) Y=5.42−0.075X1−0.018X2+0.11X3−0.096X4+0.028X1X2−9.925E−003X1X3−7.275E−003X1X4−0.026X2X3+6.825E−003X2X4−0.039X3X4−0.14X12−0.26X22−0.44X32−0.69X42.

In order to check if the model exhibited a good fit or not, regression analysis and ANOVA were necessary. According to Table 2, the corresponding coefficient of determination (Adj *R* ^2^) was 0.9296 which meant most variation (>92.96%) of the phenolic content could be predicted by the model. “Lack of fit *F*-value” of 1.57 implied the lack of fit was not significant relative to the pure error. Nonsignificant lack of fit made this model fit, so this model could be used to navigate the design space.

Values of “Prob > *F*” less than 0.05 indicated model terms were significant. In this case, the liquid to solid ratio (*X*_3_), ethanol concentration (*X*_1_), and extraction temperature (*X*_4_) were the most significant parameters which influenced the extraction yield followed by extraction time.

**Figure 2:**
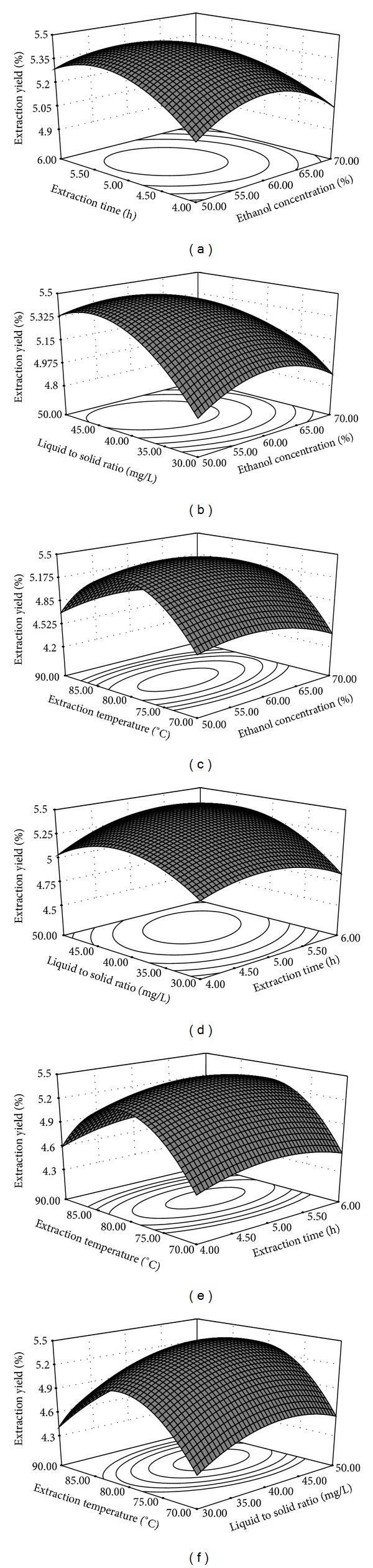
Response surface plots. Response surface plots showing the combined effects of ethanol concentration and extraction time (a), ethanol concentration and liquid to solid ratio (b), ethanol concentration and extraction temperature (c), extraction time and liquid to solid ratio (d), extraction time and extraction temperature (e), and liquid to solid ratio and extraction temperature (f).

Three-dimensional response surface plots presented in Figure 2 were accomplished by varying two variables within the experimental range under investigation, holding the other two variables at its zero level. The 3D plots demonstrated distinctly the susceptibility of response value toward the change of variable.

Figures 2(a), 2(d), and 2(e) showed the effects of extraction time and other three factors on the yield of phenolics. According to the AVONA analysis, extraction time was not a significant factor on the yield of phenolics, and the yield changed unconspicuously with the change of time when the other three factors kept at a low level. With an increase in extraction time, the yield increased to the peak when the time was up to 5 h but decreased when the concentration was higher than 5 h. This might imply choosing the right time was very vital.

The effects of ethanol concentration with each of the other factor on the yield of phenolics was presented in Figures 2(a), 2(b), and 2(c). With an increase in ethanol concentration of 50% to 57%, the yield increased to the maximum. Suitable solvent was essential to extract phenolics absolutely.

The effects of liquid to solid ratio with each of the other factors on the yield of phenolics were displayed in Figures 2(b), 2(d), and 2(f). In the three plots, the yield increased with increasing the liquid to solid ratio from 1 : 30 to 1 : 41, and thereafter decreased.

Figures 2(c), 2(e), and 2(f) showed the effects of extraction temperature with other factors on the yield of phenolics. The effects of temperature were significant and the yield changed remarkably from 70°C to 90°C. It might be interpreted as follows: the right temperature could accelerate mass transfer and improve the extraction yield.

The best extraction conditions predicted by the model were given by Expert-Design 8.06 software. The ethanol concentration of 57.17%, extraction time 4.94 h, liquid to solid ratio 1 : 41.34, and extraction temperature 79.28°C should be validated by other experiments with three replications and the predicted extraction yield was 54.46 mg/g. In order to facilitate the actual operation, we slightly modified the extraction conditions (ethanol concentration of 50%, extraction time 5 h, liquid to solid ratio 1 : 40, and extraction temperature 80°C) and verified the modified conditions were as effective as the predicted conditions (54.49 ± 4.26 mg/g), indicating the modified conditions were adequate for extraction.

### 3.2. Total Phenolic Content and Total Flavonoid Content

Total phenolic content and total flavonoid content of* Idesia polycarpa* defatted fruit residue were 54.49 ± 4.26 mgGAE/g and 23.53 ± 1.74 mgCaE/g, respectively. They were calculated, respectively, from the regression equation of calibration curves: *y* = 6.8968*x* − 0.0467  (*r* ^2^ = 0.9998) and *y* = 32.805*x* + 0.1169  (*r* ^2^ = 0.9998). In order to provide the basis for the following activity tests, the phenolics and flavonoid contents of all phase extracts were compared to each other at the same concentration (8 mg/mL). From Figure 1(f), the phenolics and flavonoid content of EAE were much higher than the other phases, which might be able to justify the excellent antioxidant effect of EAE shown in the follow-up tests.

### 3.3. Antioxidant and Depigmenting Activity

Using different accepted* in vitro* antioxidant experiments can fully analyze the* in vitro* antioxidant capacity of testing substances and thus provide some advantages for antioxidant application. DPPH method is widely used to determine lots of biological samples and food's antioxidant capacity [19]. When a free radical scavenger is added, the DPPH solution will show a gradual disappearance of the purple solution's absorption. IC_50_ of EAE (*r* ^2^ = 0.9902) presented in Table 3 was significantly less than IC_50_ of BHT (*P* < 0.01), indicating EAE had a significantly better scavenging effect than the artificially synthesized high-efficient antioxidant. The results showed the phenolics of* Idesia polycarpa* defatted residue have significant DPPH free radical scavenging activity.

ABTS free radical scavenging method has also been extensively used in the determination of total antioxidant capacity of biological samples [19]. All extracts had ABTS free radical scavenging activity and the activity increased along with the concentration increasing and there might exist a linear relationship between the concentration and the scavenging activity. The scavenging effect of EAE was still better than other extracts (Table 3). In particular, there was no significant difference of the ABTS free radical scavenging activity between 0.8 mg/mL of EAE and 0.4 mg/mL of BHT (*P* > 0.05), which suggested the extract of* Idesia polycarpa* fruit residue could have the same scavenging effect as BHT by increasing the sample concentration.

The hydroxyl radical is responsible for numerous diseases, such as oxidative damage and cell death or mutation [6]. The scavenging effect of CE and AF was several times that of EAE, while BAE had a weak scavenging ability.

**Figure 3:**
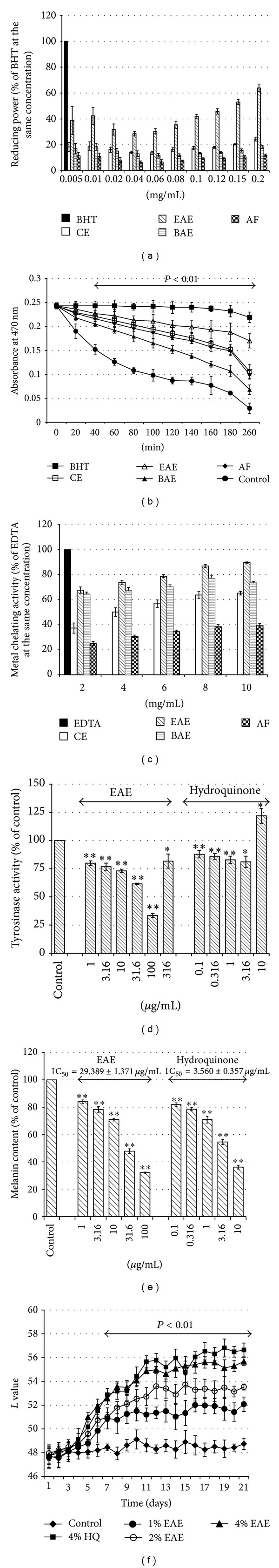
Reducing power and inhibition of *β*-carotene-linoleic acid system oxidation and depigmenting activity. (a) The reducing power of all extracts in the concentration range from 0.005 to 0.2 mg/mL. BHT was used as the positive control. (b) Inhibition of *β*-carotene-linoleic acid system oxidation by the extracts of* Idesia polycarpa* at 2 mg/mL. BHT was used as a positive control, and a negative control was completed instead of tests samples. (c) The metal chelating activity of all extracts in the concentration range from 2 to 10 mg/mL, compared to EDTA at the same concentration. (d) The tyrosinase inhibitory activity of different concentration of EAE/hydroquinone in mouse B16 melanoma cells (*P < 0.05; **P < 0.01 compared with the control). (e) The inhibitory effect of EAE and hydroquinone on melanogenesis of mouse B16 melanoma cells. Each value is expressed as mean with an error bar of standard deviation (*n* = 3). (f) Time course of skin-whitening index (*L* value) of mice; averaged *L* values (*n* = 8) with an error bar of standard deviation are plotted over the experimental period (*P* < 0.01 compared with the control group).

The material with brilliant ability of reduction could reduce Fe^3+^ into Fe^2+^ by using the color reaction to determine the extent of reduction [26]. The experiment result showed that the reducing power of BHT was more pronounced than the other four extracts (0.005 to 0.2 mg/mL, *P* < 0.05) revealed in Figure 3(a). However, as the sample concentration increased, the absorbance of each extract rose as well and showed a dose-dependent relation. EAE still had the best reducing ability among the four extracts and there was no significant difference between the absorbance of EAE at 0.2 mg/mL and BHT at 0.06 mg/mL (*P* > 0.05). The reducing power was closely linked to previous antioxidant tests, as described in the previous report [27].

Excessive O^2−^ *in vivo* is eliminated primarily by SOD. SOD is a major antioxidant enzyme* in vivo* and is widely distributed in the various biological body. SOD can catalyze superoxide radical O^2−^ and hydrogen ions react to form hydrogen peroxide and molecular oxygen and thus act as antioxidant [28]. A rapid and facile method for the assay of SOD-like activity, based on inhibiting the autooxidation of pyrogallol, is commonly used to predict antioxidant capability. Each extract of* Idesia polycarpa* could eliminate O^2−^ effectively. EAE demonstrated better performance than other three extracts at the same concentrations. All of the extracts were significantly more effective than the positive control (BHT, *P* < 0.05), but less effective than VC showed in Table 3.

*β*-carotene discoloration method mainly evaluates the antioxidant capacity of antioxidants in the emulsified lipid system [10]. Peroxide generated by the linoleic acid oxidation enables *β*-carotene bleaching in the reaction medium solution, and the absorbance at 470 nm will get smaller and smaller along with time prolonging. The absorbance curve of *β*-carotene-linoleic acid system with BHT did not show a significant decrease during 260 min presented from the Figure 3(b), while the absorbance of the blank control had fallen to 0.029. According to the research elsewhere [22], when the absorbance decreased under 0.03, *β*-carotene had seemed to be completely oxidized. The inhibitions of all extracts were 43.62%, 69.96%, 27.98%, and 40.74%, respectively, at 260 min. The absorbance of *β*-carotene-linoleic acid system with EAE declined at the slowest rate; BAE decreased at the fastest rate in comparison. Those results implied that all the extracts had the ability of terminating the lipid peroxidation chain reaction and the EAE had an admirable protective effect of linoleic acid peroxidation. Phenolics and flavonoids can inhibit the lipid peroxidation [22]. The potent inhibitory activity of EAE might be due to its high phenolics and flavonoid contents, and this could imply that EAE had a good researching value.

Lipoprotein oxidative damage is closely linked with cancer, tumor, caducity, and so forth. The inspection of lipoprotein oxidative damage may provide new clues to the study of some certain diseases [23]. The inhibitory effects of the two extracts (Table 3), CE and EAE, were markedly more efficient than *α*-tocopherol (*P* < 0.05). Moreover, the inhibition rate of EAE was 91.43 ± 1.32% at the concentration of 0.1 mg/mL and that of CE was 97.34 ± 1.08% at the concentration of 0.4 mg/mL, considerably higher than that of *α*-tocopherol (77.38 ± 1.64%) at 0.4 mg/mL.

Fe^2+^ chelating ability and antioxidant properties had a close relationship. The extracts could chelate Fe^2+^ as obviously shown in Figure 3(c), and the metal chelating activity increased in a concentration-dependent manner (2 mg/mL to 10 mg/mL). In our research, metal-chelator EDTA used as positive control was proved more powerful than the extracts (*P* < 0.05), and its chelating rate ranged from 41.95% to 68.68%, while EAE had the best chelating ability as expected and the chelating rate of EAE was equal to 89.66 ± 0.58% of that of EDTA, at 10 mg/mL.

Overall, the extracts had high total phenol and flavonoid contents. Remarkably, EAE included most of those active contents; thus we choose EAE for deeply exploring its more activities. Although there are no reports on the correlation between antioxidant activity and whitening activity, many active substances have the two activities, such as VC and EAE. Mushroom tyrosinase inhibitory activities of EAE, HQ, and arbutin were researched by using tyrosine and L-Dopa as the substrates. At the same concentration, EAE exerted qualitatively similar inhibitory effects to HQ (*P* > 0.05). Regardless of the fact that many studies have reported that arbutin could be an adequate substitute for HQ, in our experiment, arbutin did not exhibit excellent inhibitory activity as shown in Table 4. The IC_50_ of EAE had no meaningful difference from that of the HQ (*P* > 0.05), while there was a significant difference between EAE and arbutin (*P* < 0.01). Tyrosinase is essential for the production of melanin in the skin. Moreover, tyrosinase not only catalyzes harmful reactions, which deteriorated since the appearance and nutritional value of foods, but also increases the oxidative risk in different physiological systems [13]. Therefore, such tyrosinase inhibitor-active EAE would be included in general use.

Cellar tyrosinase activity and content of melanin were two notable detected indicators. HQ had distinguished inhibitory activity in the production of melanin, and distinct cytotoxicity of B16 cells. From Table 4, both of EAE and HQ exhibited inhibition of B16 cell proliferation but also showed significant melanogenesis inhibitory activities. The cytotoxicity of HQ was 10.5 times more venomous than that of EAE, while the melanogenesis inhibitory activity of HQ was 8.3 times higher than that of EAE (Figure 3(e)). The results of cell experiments showed EAE strongly inhibited cellular tyrosinase activity, which might be the most reliable reason why it could lead to the decrease of melanogenesis in B16 mouse melanoma cells.

As shown in Figure 3(d), EAE showed a strong inhibitory tyrosinase activity, but the inhibitory activity of HQ was weak. Inhibitory activity of EAE became stronger with the EAE's concentration increasing, which displayed the dose-effect relationship. 316 *μ*g/mL of EAE and 100 *μ*g/mL of HQ exhibited intense inhibition of B16 cell proliferation, survival of cells was scarce, and that was the reason why the relative inhibitory activity much weakened. The inhibitory activity of idescarpin separated and identified from EAE for melanin synthesis has been confirmed [2]. Idesin and idescarpin have a similar structure [3]. Whether idesin has similar whitening activity needs to be further verified.

In order to simulate more closely approximate conditions of human skin, we used C57BL/6 mice as the animal model for exploring the depigmenting activity of EAE. Even though the depigmenting activity of HQ was obviously excellent for all, 1%, 2%, and 4% of EAE showed notable activity, compared to the control as shown in Figure 3(f). Moreover, the depigmenting activity of 4% of EAE was nearly similar to that of 4% of HQ in the last stage of the experiment. Nevertheless, other than that, the daily actions of the smeared mice were all going well, and the skins had no macroscopic hypersensitivity and fester situation. EAE administrated by smearing twice daily could significantly brighten skin, which might imply EAE has the enormous potential for being developed as a new natural skin-lightening agent.

## 4. Conclusion

This study established an extraction procedure and assessed the potential antioxidant and depigmenting activity* in vitro* and* in vivo* for phenolics from* Idesia polycarpa* defatted fruit residue. As far as we know, this is the first report on the extraction optimization of phenolics from* Idesia polycarpa* using RSM and also the first time the antioxidant and* in vivo* depigmenting activity of EAE is studied. High phenolics content and excellent antioxidant and depigmenting activity make it believable that* Idesia polycarpa* fruit has the development potential for antioxidant and depigmenting properties extraction and can thus be applied to cosmetics, food additives, and medicine.

## Figures and Tables

**Table 1:** Box-Behnken design and observed responses.

Run	*X*_1_: ethanol concentration (%)	*X*_2_: extraction time (h)	*X*_3_: liquid to solid ratio (mg/L)	*X*_4_: extraction temperature (°C)	Response: extraction yield (%)^a^
1	50.00	4.00	40.00	80.00	5.2221
2	70.00	4.00	40.00	80.00	4.9714
3	50.00	6.00	40.00	80.00	5.1743
4	70.00	6.00	40.00	80.00	5.0347
5	60.00	5.00	30.00	70.00	4.3575
6	60.00	5.00	50.00	70.00	4.5147
7	60.00	5.00	30.00	90.00	4.2877
8	60.00	5.00	50.00	90.00	4.2887
9	50.00	5.00	40.00	70.00	4.7512
10	70.00	5.00	40.00	70.00	4.6228
11	50.00	5.00	40.00	90.00	4.5787
12	70.00	5.00	40.00	90.00	4.4212
13	60.00	4.00	30.00	80.00	4.6159
14	60.00	6.00	30.00	80.00	4.61
15	60.00	4.00	50.00	80.00	4.9015
16	60.00	6.00	50.00	80.00	4.7915
17	50.00	5.00	30.00	80.00	4.6551
18	70.00	5.00	30.00	80.00	4.5633
19	50.00	5.00	50.00	80.00	5.0227
20	70.00	5.00	50.00	80.00	4.8912
21	60.00	4.00	40.00	70.00	4.5651
22	60.00	6.00	40.00	70.00	4.4915
23	60.00	4.00	40.00	90.00	4.3118
24	60.00	6.00	40.00	90.00	4.2655
25	60.00	5.00	40.00	80.00	5.4548
26	60.00	5.00	40.00	80.00	5.5487
27	60.00	5.00	40.00	80.00	5.3117
28	60.00	5.00	40.00	80.00	5.4224
29	60.00	5.00	40.00	80.00	5.3845

^a^Phenolics extraction yield is percentage content of the extracted phenolics in the studied raw material.

**Table 2:** ANOVA for the effect of ethanol concentration, liquid to solid ratio, temperature, and time on the yield of phenolics using a quadratic response surface model.

Source	SS^a^	df^b^	MS^c^	*F* ratio	*P* value
Model	4.15	14	0.30	27.40	<0.0001
*X*_1_	0.067	1	0.067	6.24	0.0256
*X*_2_	4.044*E* − 003	1	4.044*E* − 003	0.37	0.5506
*X*_3_	0.15	1	0.15	13.44	0.0025
*X*_4_	0.11	1	0.11	10.18	0.0065
*X*_1_ *X*_2_	3.086*E* − 003	1	3.086*E* − 003	0.29	0.6016
*X*_1_ *X*_3_	3.940*E* − 004	1	3.940*E* − 004	0.036	0.8513
*X*_1_ *X*_4_	2.117*E* − 004	1	2.117*E* − 004	0.020	0.8907
*X*_2_ *X*_3_	2.709*E* − 003	1	2.709*E* − 003	0.25	0.6245
*X*_2_ *X*_4_	1.863*E* − 004	1	1.863*E* − 004	0.017	0.8974
*X*_3_ *X*_4_	6.100*E* − 003	1	6.100*E* − 003	0.56	0.4650
*X*_1_^2^	0.12	1	0.12	11.18	0.0048
*X*_2_^2^	0.42	1	0.42	39.23	<0.0001
*X*_3_^2^	1.24	1	1.24	114.91	<0.0001
*X*_4_^2^	3.12	1	3.12	288.17	<0.0001
Residual	0.15	14	0.011		
Lack of fit	0.12	10	0.012	1.57	0.3511
Pure error	0.031	4	7.668*E* − 003		
*R* ^2^	0.9648				
Adj *R* ^2^	0.9296				

^a^Sum of squares, ^b^mean square, and ^c^degree of freedom.

**Table 3:** DPPH, ABTS, and hydroxyl free radicals scavenging activities and inhibition of lipoprotein oxidation and SOD-like activities with IC_50 _values of the extracts of *Idesia polycarpa* fruit and BHT (or AA, VE) as positive control.

Sample	DPPH radical^a^	ABTS radical^a^	Hydroxyl radical^a^	Lipoprotein^a^	SOD-like activity^a^ (%) at 0.1 mg/mL	SOD-like activity^a^ (%) at 0.2 mg/mL
	IC_50_^b^ (mg/mL)	IC_50_^b^ (mg/mL)	IC_50_^b^ (mg/mL)	IC_50_^b^ (mg/mL)		
CE	0.093 ± 0.007**	0.43 ± 0.04**	2.08 ± 0.08**	0.085 ± 0.007*	49.33 ± 0.93*	54.92 ± 0.63**
EAE	0.032 ± 0.002**	0.35 ± 0.03**	7.20 ± 0.18**	0.065 ± 0.001**	60.00 ± 0.78**	65.27 ± 0.39**
BAE	0.131 ± 0.020**	0.64 ± 0.10**	—	0.353 ± 0.024**	54.22 ± 0.39**	59.94 ± 0.54**
AF	0.213 ± 0.030**	0.74 ± 0.04**	2.06 ± 0.20**	0.815 ± 0.065**	47.43 ± 0.97	54.09 ± 1.02**
BHT	0.067 ± 0.004	0.091 ± 0.008	—	—	45.72 ± 2.19	47.05 ± 2.35
VE/AA	—	—	0.095 ± 0.003	0.114 ± 0.016^c^	96.24 ± 0.86^c∗∗^	99.11 ± 0.32^c∗∗^

^a^Each value is expressed as mean ± standard deviation (*n* = 3).

^b^IC_50_ (mg/mL): the concentration at which 50% is inhibited.

^c^VE as the control.

∗ *P* < 0.05; ∗∗*P* < 0.01 compared with BHT.

**Table 4:** Effects of EAE against mushroom tyrosinase and on cell growth of B16 cells.

Sample	Mushroom tyrosinase IC_50_ (tyrosine, mg/mL)	Mushroom tyrosinase IC_50_ (L-Dopa, mg/mL)	Cytotoxicities LD_50_ (g/mL)^a^
EAE	1.009 ± 0.046	2.122 ± 0.106	29.407 ± 0.381
Hydroquinone	0.983 ± 0.032	2.173 ± 0.188	2.798 ± 0.106
Arbutin	1.407 ± 0.067^A^	3.5994 ± 0.193^A^	—

^a^LD_50_: 50% lethal dose.

^A^ *P* < 0.01 compared with EAE.

## Abbreviations

FC-reagent: Folin-Ciocalteu reagent
TBA: Thiobarbituric acid
ABTS: 2,2′-Azinobis-(3-ethylbenzthiazoline-6-sulphonate)
DPPH: 1,1-Diphenyl-2-picrylhydrazyl radical
BHT: Butylated hydroxyl toluene
VE: *α*-Tocopherol
VC: Ascorbic acid
MTT: 3-(4,5-Dimethylthiazol-2-yl)-2,5-diphenyltetrazolium bromide
HQ: Hydroquinone
CE: Crude extract
EAE: Ethyl acetate extraction
BAE: n-Butyl alcohol extraction
AF: Aqueous fraction
RSM: Response surface methodology
TCA: Trichloroacetic acid
TBA: Thiobarbituric acid
IC_50_: Half-maximal inhibitory concentration.
